# Histopathological Findings of Failed Free Vascularized Fibular Grafting for Osteonecrosis of the Femoral Head after Long-Term Follow-Up

**DOI:** 10.1155/2020/6493585

**Published:** 2020-06-14

**Authors:** Thou Lim, Qian Tang, Qiyang Wang, Zhenzhong Zhu, Xiaojuan Wei, Yong Feng, Changqing Zhang

**Affiliations:** ^1^Department of Orthopedic Surgery, Shanghai Jiao Tong University Affiliated Sixth People's Hospital, 600 Yishan Road, Shanghai 200233, China; ^2^Institute of Microsurgery on Extremities, Shanghai Jiao Tong University Affiliated Sixth People's Hospital, 600 Yishan Road, Shanghai 200233, China

## Abstract

**Purpose:**

The aim of this study was to report the histopathology of failed free vascularized fibular grafting (FVFG) for osteonecrosis of the femoral head (ONFH) after a mean follow-up of 11.5 years (ranged from 10.6 to 14.2 years).

**Methods:**

Six hips of 5 patients with a history of steroid use, aged 34–67 years, were in stage II of ONFH as classified by the Ficat and Arlet classification at the time of FVFG treatment. Grafting failure led to osteoarthritis of the hip joint during a mean of 11.5 years of follow-up. Femoral head specimens were first evaluated macroscopically. Bone specimens were sectioned into long strips, divided into necrotic, transitional, and healthy zones, and then prepared for nondecalcified and decalcified histopathological examination using hematoxylin and eosin (HE) staining, Goldner's trichrome staining, and immunofluorescence (IF) staining.

**Results:**

Femoral head articular cartilage surfaces appeared thin, opaque, and partially cartilaginous missing, with gradual collapse detected in weight-bearing areas. The interface with the fibular graft showed well union, with no obvious gaps between graft and host bone, as observed macroscopically. The necrotic area was filled with fibular graft, cancellous bone, and cartilaginous or soft tissue invasion. Histopathology results revealed well integration between fibular graft and host bone, with thickened trabecular bone. Gaps occurred in transitional and healthy zones. In the necrotic zone, cartilaginous or soft tissue invasion occurred, while thin or missing articular cartilage exposed subchondral bone to hip joint surfaces. By IF counterstaining with CD-31 and *α*-SMA, blood vessel transplanted during fibular grafting could be clearly observed along the graft from healthy to necrotic zones. In the necrotic zone, blood vessels presented obviously and spread into the surrounding area of the graft tip.

**Conclusion:**

After FVFG procedure with a mean follow-up of 11.5 years, fibular grafts retained their integrity as viable, vascularized, cortical bone that fused well with host bone and formed thickened trabecular bone surrounding the surface of the graft. However, the revascularization of FVFG's blood vessels spreading from the tip of the fibular graft into subchondral area of necrotic lesion did not improve significantly in these failure cases. The local necrotic lesion failed to be repaired as healthy trabecular bone to buttress articular surface and was occupied by soft tissues.

## 1. Introduction

Osteonecrosis of the femoral head (ONFH) is a progressive pathological process caused by disruption of the blood supply in the femoral head, leading to collapse of the articular surface and subsequent osteoarthritis (OA) of the hip joint. This disabling disease may originate from trauma (e.g., hip dislocation or femoral neck fracture) or from nontraumatic causes (e.g., steroid use, alcohol abuse, autoimmune disease, sickle cell disease, or idiopathic osteonecrosis). Nontraumatic ONFH typically affects patients between the ages of 30 and 50 years and progresses from early to end stages, causing collapse of the femoral head in 80% of untreated patients [1–3]. Early diagnosis is extremely important to halt or reverse the progression of the disease, and stage at time of treatment is a major factor in the success or failure of surgical procedures for hip joint preservation [2]. Reports have indicated that joint preservation procedures can achieve good outcomes if employed early, before collapse of the hip joint, whereas those performed after collapse of the femoral head lead to early failure [4–8].

Over the last decades, several procedures have been used to treat ONFH, primarily core decompression [7], transtrochanteric rotation osteotomy [9], electrical stimulation [10], and free vascularized [11] and nonvascularized [12] fibular grafting. Free vascularized fibular grafting (FVFG) is considered effective for patients with early-stage ONFH. FVFG is believed to not only provide support to buttress the articular surface but also to revascularize the necrotic lesion, restoring blood supply to the femoral head. Moreover, FVFG is believed to carry bone mesenchymal stem cells into the necrotic area [6, 13, 14]. However, the failure rate of FVFG ranges from 4 to 30%, as reported in previous studies [15, 16]. Failure leads to further progression of ONFH, causing collapse of the articular surface and persistent hip joint pain warranting total hip arthroplasty (THA). To date, the causes of failure are still unclear. The aim of this study was to report the histopathology of failed FVFG for ONFH after a mean follow-up of 11.5 years.

## 2. Materials and Methods

### 2.1. Surgical Technique

The initial FVFG procedure performed is as described previously [17]. Briefly, the approach to the hip was through the Smith-Peterson incision. After the incision of the fascia lata, the space between the tensor fasciae latae and sartorius was exposed. The tendon of rectus femoris muscle was partially transected to the anterior inferior iliac spine, where the rectus femoris is attached. At the anterior margin of the acetabulum, the reflected head of the rectus femoris was cut. After the rectus femoris was turned over, the lateral femoral circumflex artery and vein were exposed to serve as recipient vessels for FVFG. A longitudinal capsulotomy was performed to expose the femoral neck. Then, a bone groove was made with the size to match fibula graft at the anterior aspect of the femoral neck by bone chisel. The groove was extended to the center of necrotic foci. An additional canal, which was made from the great tuberosity face to the femoral head, was made with a drill. A burr was used to remove the necrotic tissue from the femoral head with 5 mm beneath the cartilage surface through the bone groove. The cancellous bone chips from the groove creation were used to fill the cavity in the femoral head. The fibula graft was inserted in the groove (the vessels should be placed at the anterior aspect). The graft fibula was stabilized to the femoral neck with an absorbable screw. Under an operating microscope, arterial and venous anastomoses were performed with 6-0 interrupted nylon sutures. Bleeding from the cortical bone at the base of the fibular graft confirmed the vascularity of the graft.

### 2.2. Specimens Harvested

**Figure 1:**
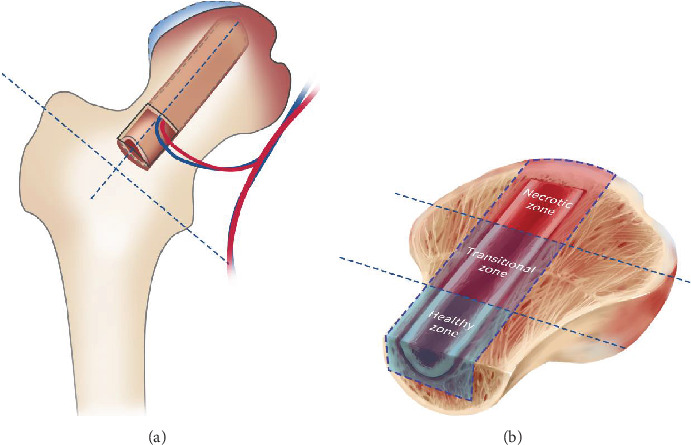
Schematic of failed FVFG femoral head. (a) Osteotomy of failed FVFG femoral head during THA procedure. (b) The upper half of the femoral head was divided into three interested zones: necrotic, transitional, and healthy zones.

This study was carried out in accordance with the Helsinki Declaration. All specimens were obtained during THA revision surgery, and each patient provided written informed consent, as approved by the ethical committee of the Shanghai Jiao Tong University Affiliated Sixth People's Hospital. FVFG preoperative medical records (patients' radiographs, stage at time of grafting by the Ficat and Arlet classification, and history of steroid use) and FVFG postoperative data (follow-up radiographs and time interval between grafting to conversion THA surgery) were obtained. During 2004 to 2008, there were 950 cases of ONFH patients who underwent FVFG procedure in our center. We collected failure FVFG samples diagnosed with ONFH in stage II classified by the Ficat and Arlet classification, with a history of steroid use. Since ONFH may originate from many causes and the progression of ONFH will appear in different stages, therefore, these conditions are considered as variables in this study. In order to reduce variables, the design is to observe the same criteria of diagnosis and staging of ONFH in failure cases. After exclusion of patients with stage I, III, and IV ONFH and of those with ONFH from other causes, there were six hips of stage II ONFH with histories of steroid use and of persistent pain after failure of FVFG procedures requiring conversion THA. One male and four female patients (one female patient with failure of bilateral FVFG), aged 34–67 years (mean 51.5 years), were included in this study. The time interval between FVFG and THA ranged from 10.6 to 14.2 years (mean 11.5 years). Failed FVFG specimens were collected by osteotomy of the femoral head during THA procedures (Figure 1(a)).

### 2.3. Gross Observation

All specimens were examined for the presence of abnormal contours, collapse, and lipping, and the condition of the articular cartilage surface (degeneration, thin or missing in weight-bearing areas) was noted. The specimens were cut into upper and lower parts, along the long axis of the implanted bone graft, immediately following osteotomy of the femoral head during THA. The specimens were observed macroscopically to evaluate the state of repairing in necrotic areas and the overall integrity of interfaces between graft and host bone.

### 2.4. Specimens' Preparation

Our study focused on investigating the upper part of specimens located in weight-bearing areas of necrotic lesions. Each specimen was first cut into a long strip, consisting of the upper part of the fibular graft and host bone, leaving at least a 3 mm thickness of cancellous bone from the graft, circumferentially. This strip segment was further divided into three parts: a necrotic zone (the graft segment located from the proximal half of the femoral head to the cartilage surface), a transitional zone (the middle part of the segment, in the distal half of the femoral head), and a healthy zone (the lower part of the segment located in the femoral neck area) (Figure 1(b)). Both decalcified and nondecalcified segments were prepared from all specimens.

For nondecalcified preparations, specimens were dehydrated through a graded alcohol series, immersed in xylene, and embedded in polymethyl methacrylate under negative pressure. After solidification, 100 *μ*m thick sections were cut using a saw microtome, glued onto a transparent plastic plate, and stained with hematoxylin and eosin (HE). Sections, 10 *μ*m thick, were stained by Goldner's trichrome method in order to distinguish between osteoid, mineralized bone, and cellular components in three interested zones and examined by light microscopy.

For decalcified preparations, specimens were decalcified in 10% ethylenediaminetetraacetic acid (EDTA) for 12 weeks, dehydrated in a graded alcohol series, embedded in paraffin, sectioned into 5 *μ*m thick sections, stained with HE, and examined by light microscopy.

For IF staining, decalcified sections were rehydrated, blocked with 1% bovine serum albumin for 30 min at room temperature, incubated in primary antibodies against CD31 (1 : 100, Abcam) and *α*-SMA (1 : 100, Abcam) at 4°C overnight, then incubated with Alexa Fluor 488- and Cy3-conjugated secondary antibodies for 1 h at room temperature and counterstained with DAPI. Images were acquired with a LEICA DM 4000 fluorescence microscope.

## 3. Results

### 3.1. Patients' Demographics and Radiographs

**Figure 2:**
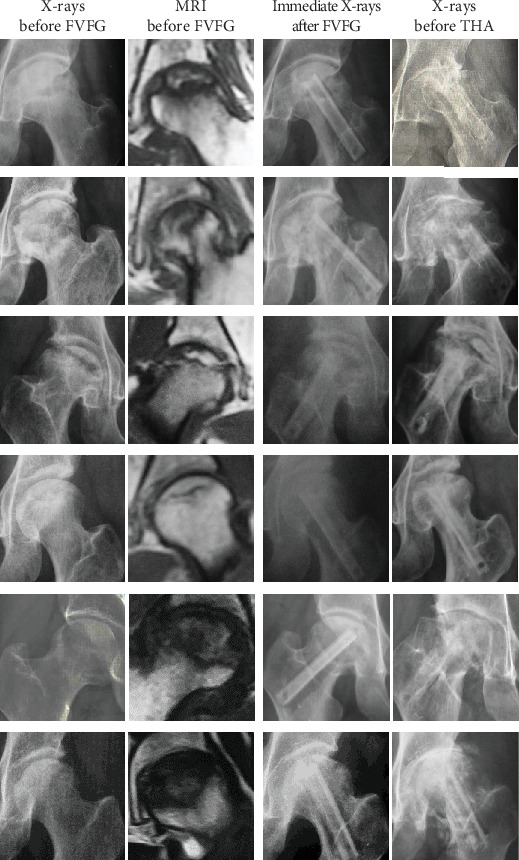
Six failure cases' radiographs (X-rays and MRI before FVFG, immediate X-rays after FVFG, and X-ray before THA).

Patients' medical records (gender, age at the time of initial FVFG procedure and THA revision surgery, diagnosis, X-rays, and MRI) were collected, as shown in Table 1 and Figure 2.

### 3.2. Gross Observation

**Figure 3:**
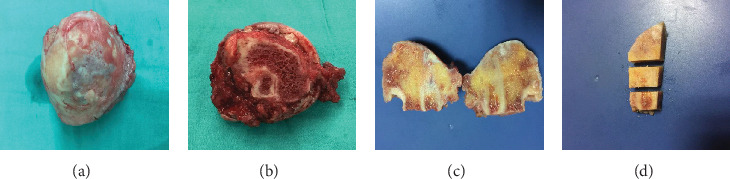
The macroscopic view of failed FVFG femoral head and the three interested zones. (a, b) Macroscopic view of failed FVFG femoral head. (c) Cutting surface macroscopic view of FVFG. (d) Three interested zones in the upper half of the femoral head.

In the six failure cases, gross observation of the articular surface in the fresh specimens showed gradual collapse in the weight-bearing area, thin or missing cartilage surfaces, exposing subchondral bone, surrounded by degenerated articular cartilage. The articular cartilage overall lost its normal hyaline appearance, and the surfaces were roughened and opaque. Around the edges of articular surfaces, cartilage lipping and osteophyte formation were noted. The interface with the fibular graft showed well union, with no obvious gaps between graft and host bone, as observed macroscopically. The grafts remained intact and well fused to the host bone. The necrotic area was filled with fibular graft, cancellous bone, and cartilaginous or soft tissue invasion (Figures 3(a)–3(d)).

### 3.3. Histopathology

**Figure 4:**
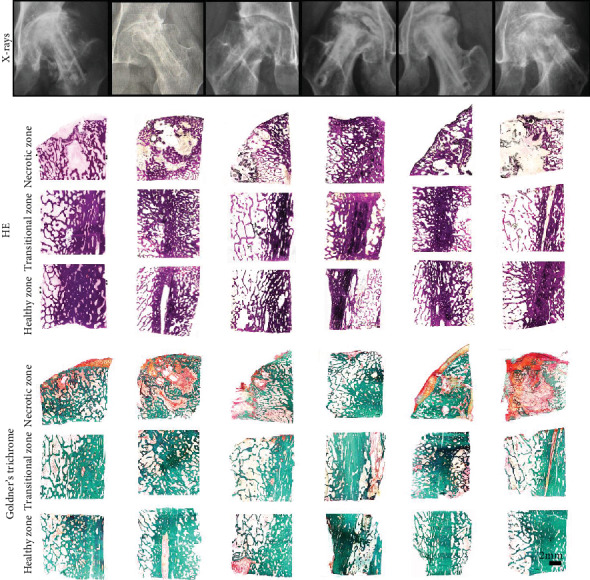
Radiographs and histology (HE and Goldner's trichrome staining of three interested zones) of six failure cases.

HE and Goldner's trichrome staining of nondecalcified specimens showed good integration between fibular graft and host bone, with dense trabecular bone surrounding the surfaces of the fibular graft in three zones. Gaps occurred in the transitional and healthy zones. Thin or missing articular cartilage exposed subchondral bone to hip joint surfaces. Cartilaginous or soft tissue invasion occurred evidently in the necrotic zone, as compared to the transitional and healthy zones (Figure 4).

**Figure 5:**
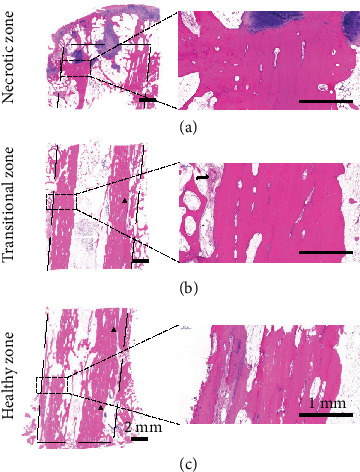
Decalcified HE staining of three interested zones. Gaps (black triangles) appeared in the healthy and transitional zones; blood vessel (black arrow) appeared along the graft implanted. Scale bars 2 mm and 1 mm.

Decalcified paraffin-embedded HE-stained sections showed well-integrated graft with the host bone. Gaps were found in the transitional and healthy zones (black triangles, Figure 5). The articular cartilage was thin or partially missing, exposing subchondral plate beneath to the hip joint. Subchondral bone plate was thin. Around the tip of the fibular graft, the invasion of chondral tissue could be observed. The fibular graft was viable, as evidenced by the presence of stainable osteocyte nuclei in the lacunae of the bone graft in three zones. With HE staining, blood vessels could be detected, located along the surface of the graft (black arrow, Figure 5).

### 3.4. Immunofluorescence Staining

**Figure 6:**
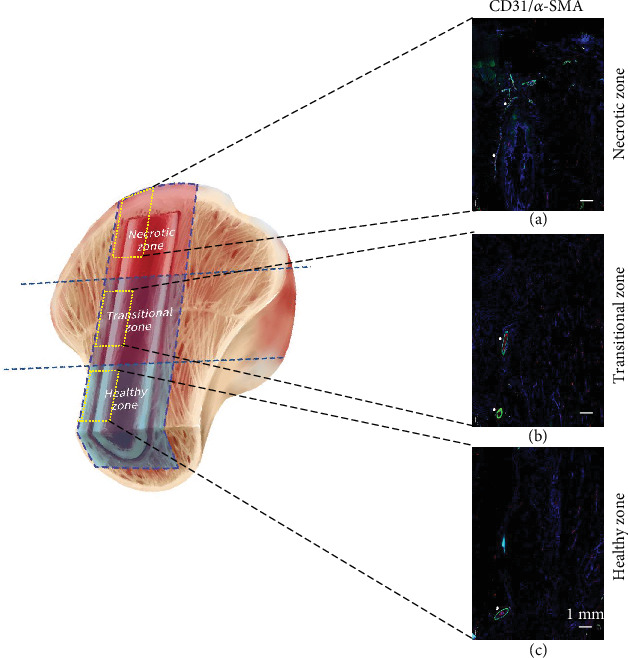
IF counterstaining with CD-31 and *α*-SMA of blood vessels in the three zones of interest: (a) necrotic zone, (b) transitional zone, and (c) healthy zone. White triangles indicated blood vessels along the implanted bone graft in the three zones of interest.

Implanted grafts were viable, as demonstrated by the presence of DAPI-stained nuclei in the three zones. By IF counterstaining with CD-31 and *α*-SMA, blood vessel transplanted during fibular grafting could be clearly observed along the graft from the healthy zone to the necrotic zone after implanted for the mean period of 11.5 years (white triangles, Figures 6(a)–6(c)). Blood vessels were stained in the tip of the graft and spread into the surrounding area around the tip of the graft in the necrotic zone (Figure 6(a)).

## 4. Discussion

Free vascularized fibular grafting is a procedure aimed at replacing the necrotic bone that has lost its mechanical function with viable cortical bone, buttressing the articular surface, and revascularizing the necrotic lesion and forming new bone, preventing collapse. Clearance of necrotic lesion, filling the necrotic space with the implanted bone graft and restoring blood supply, creates conditions for the formation of the new trabecular bone needed to effectively restore mechanical loading to necrotic areas and in order to achieve joint preservation after ONFH. Revascularization of the fibular graft should enhance its incorporation into the host, replacing the necrotic core bone with new, viable bone [11, 18, 19].

Properly locating graft insertions in the necrotic area is essential to support the subchondral plate and revascularization, as has been suggested by other authors. Beris and Soucacos [20] suggested that proper placement of the bone graft to buttress the subchondral plate might be the most important factor in preventing subchondral collapse. Gonzalez Della Valle et al. [21] concluded that FVFG failure was the consequence of improper placement, in which the graft did not provide mechanical support to the subchondral plate.

After FVFG procedures with a mean follow-up of 11.5 years, fibular grafts retained their integrity as viable, vascularized, cortical bone. We observed positive changes after FVFG procedure of which the bone graft was effectively fused with the host bone and formed thickened trabecular bone surrounding the surface of the graft. However, the repair of necrotic bone to form healthy trabecular bone related to revascularized condition to bring blood supply into the necrotic lesion. According to the results of IF staining, the revascularization of FVFG's blood vessels spreading from the tip of the fibular graft into the subchondral area of necrotic lesion did not improve significantly in these failure cases. The loss of local blood supply might fail to repair the necrotic lesion, which resulted in the absorbance of the necrotic bone. The lesion then failed to be repaired as healthy trabecular bone to buttress articular surface and was occupied by soft tissues.

In our findings, gaps occurred in the defined transitional and healthy zones. This was similar to the observations of Gonzalez Della Valle et al. [21], who reported that union was mostly present in the cancellous bone of the head, with gaps occurring in the femoral neck. By contrast, Meloni et al. [22] reported that the most striking osteoformative reaction to the graft was in the neck and was the lowest in the necrotic area.

Our findings are based on six FVFG failure cases with a history of steroid use and stage II ONFH; more case series in other cause factors and stages are needed to be further studied.

## 5. Conclusion

After FVFG procedure with a mean follow-up of 11.5 years, fibular grafts retained their integrity as viable, vascularized, cortical bone that fused well with the host bone and formed thickened trabecular bone surrounding the surface of the graft. However, the revascularization of FVFG's blood vessels spreading from the tip of the fibular graft into the subchondral area of necrotic lesion did not improve significantly in these failure cases. The local necrotic lesion failed to be repaired as healthy trabecular bone to buttress articular surface and was occupied by soft tissues.

## Figures and Tables

**Table 1:** Details of six failure cases.

Patients' gender	Patients' age at FVFG	Diagnosis	Size of lesion by MRI	Patients' age at THA
M	53	ONFH (left, stage II, steroid)	47.90%	63
F	46	ONFH (left, stage II, steroid)	72.73%	57
F	53	ONFH (left, stage II, steroid)	71.58%	67
F	23	ONFH (right, stage II, steroid)	62.59%	34
F	34	ONFH (left, stage II, steroid)	19.68%	44
F	34	ONFH (right, stage II, steroid)	32.63%	44

## Data Availability

The data used to support the findings of this study are included within the article.

## References

[1] Fukushima W., Fujioka M., Kubo T., Tamakoshi A., Nagai M., Hirota Y. (2010). Nationwide epidemiologic survey of idiopathic osteonecrosis of the femoral head. *Clinical Orthopaedics and Related Research*.

[2] Mont M. A., Hungerford D. S. (1995). Non-traumatic avascular necrosis of the femoral head. *The Journal of Bone and Joint Surgery American*.

[3] Lieberman J. R., Berry D. J., Mont M. A. (2003). Osteonecrosis of the hip: management in the 21st century. *Instructional Course Lectures*.

[4] Malizos K. N., Quarles L. D., Dailiana Z. H., Rizk W. S., Seaber A. V., Urbaniak J. R. (2004). Analysis of failures after vascularized fibular grafting in femoral head necrosis. *The Orthopedic Clinics of North America*.

[5] Lavernia C. J., Sierra R. J., Grieco F. R. (1999). Osteonecrosis of the femoral head. *The Journal of the American Academy of Orthopaedic Surgeons*.

[6] Scully S. P., Aaron R. K., Urbaniak J. R. (1998). Survival analysis of hips treated with core decompression or vascularized fibular grafting because of avascular necrosis. *The Journal of Bone and Joint Surgery American*.

[7] Fairbank A. C., Bhatia D., Jinnah R. H., Hungerford D. S. (1995). Long-term results of core decompression for ischaemic necrosis of the femoral head. *Journal of Bone and Joint Surgery British*.

[8] Bozic K. J., Zurakowski D., Thornhill T. S. (1999). Survivorship analysis of hips treated with core decompression for nontraumatic osteonecrosis of the femoral head. *The Journal of Bone and Joint Surgery American*.

[9] Scher M. A., Jakim I. (1993). Intertrochanteric osteotomy and autogenous bone-grafting for avascular necrosis of the femoral head. *The Journal of Bone and Joint Surgery American*.

[10] Steinberg M. E., Brighton C. T., Corces A. (1989). Osteonecrosis of the femoral head. Results of core decompression and grafting with and without electrical stimulation. *Clinical Orthopaedics and Related Research*.

[11] Brunelli G., Vigasio A., Battiston B., di Rosa F., Brunelli G Jr (1991). Free microvascular fibular versus conventional bone grafts. *International Surgery*.

[12] Buckley P. D., Gearen P. F., Petty R. W. (1991). Structural bone-grafting for early atraumatic avascular necrosis of the femoral head. *The Journal of Bone and Joint Surgery American*.

[13] Soucacos P. N., Beris A. E., Malizos K., Koropilias A., Zalavras H., Dailiana Z. (2001). Treatment of avascular necrosis of the femoral head with vascularized fibular transplant. *Clinical Orthopaedics and Related Research*.

[14] Korompilias A. V., Beris A. E., Lykissas M. G., Kostas-Agnantis I. P., Soucacos P. N. (2011). Femoral head osteonecrosis: why choose free vascularized fibula grafting. *Microsurgery*.

[15] Urbaniak J. R., Coogan P. G., Gunneson E. B., Nunley J. A. (1995). Treatment of osteonecrosis of the femoral head with free vascularized fibular grafting. A long-term follow-up study of one hundred and three hips. *The Journal of Bone and Joint Surgery American*.

[16] Zhang C., Zeng B., Xu Z. (2004). Treatment of osteonecrosis of femoral head with free vascularized fibula grafting. *Zhongguo Xiu Fu Chong Jian Wai Ke Za Zhi*.

[17] Zhang C., Zeng B., Xu Z. (2005). Treatment of femoral head necrosis with free vascularized fibula grafting: a preliminary report. *Microsurgery*.

[18] Gonzalez del Pino J., Knapp K., Gomez Castresana F., Benito M. (1990). Revascularization of femoral head ischemic necrosis with vascularized bone graft: a CT scan experimental study. *Skeletal Radiology*.

[19] Gilbert A., Judet H., Judet J., Ayatti A. (1986). Microvascular transfer of the fibula for necrosis of the femoral head. *Orthopedics*.

[20] Beris A. E., Soucacos P. N. (2001). Optimizing free fibular grafting in femoral head osteonecrosis: the Ioannina aiming device. *Clinical Orthopaedics and Related Research*.

[21] Gonzalez Della Valle A., Bates J., Di Carlo E., Salvati E. A. (2005). Failure of free vascularized fibular graft for osteonecrosis of the femoral head: a histopathologic study of 6 cases. *The Journal of Arthroplasty*.

[22] Meloni M. C., Hoedemaeker W. R., Fornasier V. (2016). Failed vascularized fibular graft in treatment of osteonecrosis of the femoral head. A histopathological analysis. *Joints*.

